# Urine-Derived Epithelial Cells as a New Model to Study Renal Metabolic Phenotypes of Patients with Glycogen Storage Disease 1a

**DOI:** 10.3390/ijms24010232

**Published:** 2022-12-23

**Authors:** Livia Lenzini, Elisabetta Iori, Federico Scannapieco, Gianni Carraro, Angelo Avogaro, Nicola Vitturi

**Affiliations:** 1 Emergency Medicine Unit and Specialized Center of Excellence for Hypertension of the European Society of Hypertension, Department of Medicine-DIMED, University Hospital, 35128 Padova, Italy; 2 Division of Metabolic Diseases, Department of Medicine-DIMED, University Hospital, 35128 Padova, Italy; 3 Nephrology, Dialysis and Transplant Unit, Department of Medicine-DIMED, University Hospital, 35128 Padova, Italy

**Keywords:** glycogen storage disease, urine-derived cells, reactive oxygen species

## Abstract

Glycogen storage diseases (GSDs) represent a model of pathological accumulation of glycogen disease in the kidney that, in animal models, results in nephropathy due to abnormal autophagy and mitochondrial function. Patients with Glycogen Storage Disease 1a (GSD1a) accumulate glycogen in the kidneys and suffer a disease resembling diabetic nephropathy that can progress to renal failure. In this study, we addressed whether urine-derived epithelial cells (URECs) from patients with GSD1a maintain their biological features, and whether they can be used as a model to study the renal and metabolic phenotypes of this genetic condition. Studies were performed on cells extracted from urine samples of GSD1a and healthy subjects. URECs were characterized after the fourth passage by transmission electron microscopy and immunofluorescence. Reactive oxygen species (ROS), at different glucose concentrations, were measured by fluorescent staining. We cultured URECs from three patients with GSD1a and three healthy controls. At the fourth passage, URECs from GSD1a patients maintained their massive glycogen content. GSD1a and control cells showed the ciliary structures of renal tubular epithelium and the expression of epithelial (E-cadherin) and renal tubular cells (aquaporin 1 and 2) markers. Moreover, URECs from both groups responded to changes in glucose concentrations by modulating ROS levels. GSD1a cells were featured by a specific response to the low glucose stimulus, which is the condition that more resembles the metabolic derangement of patients with GSD1a. Through this study, we demonstrated that URECs might represent a promising experimental model to study the molecular mechanisms leading to renal damage in GSD1a, due to pathological glycogen storage.

## 1. Introduction

Glycogen Storage Disease 1a (GSD1a) (OMIM: 232200) is a disorder of glycogen metabolism caused by a loss of function of glucose-6-phosphatase (G6Pase) α [1]. The enzyme, expressed in the liver, kidney, and intestine, modifies glucose-6-phosphate at the terminal step of gluconeogenesis and glycogenolysis [2].

GSD1a patients accumulate glycogen not only in the liver, but also in the kidneys, and suffer a disease resembling diabetic nephropathy that can progress to renal failure [3]. Most of the GSD1a patients above 25 years of age exhibit microalbuminuria and more than 50% proteinuria [4]. Moreover, in animal models with renal-targeted deletion of G6Pase, early-onset nephropathy was observed [5], with an increase in oxidative stress levels due to the activation of the angiotensin II and TGF-b1 pathways [6].

However, despite developing many animal models of GSD1a [5,7,8,9,10,11], only one human cell model of GSD1a has been reported [12], and it did not focus on kidney cells to study the molecular mechanisms involved in kidney disease in this condition.

In this study, we addressed whether urine-derived epithelial cells (URECs) from patients with GSD1a can be used as a model to study the renal metabolic phenotypes of this rare genetic disease. Since kidney oxidative stress was reported in mice with renal-targeted deletion of G6Pase [6], as the first proof-of-concept study, we tested the capability of this cell model to respond to a metabolic stimulus (glucose) by modulating reactive oxidative species levels.

## 2. Results

### 2.1. Characteristics of URECs

In this study, we obtained sufficient amounts of cells in three samples from GSD1a patients (Table 1) and three healthy age-matched controls.

**Figure 1:**
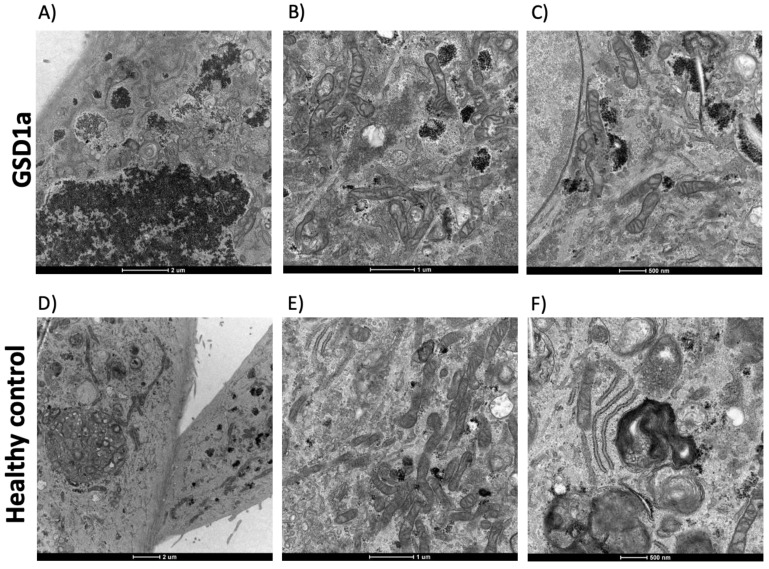
Transmission Electron Microscopy (TEM) was used to provide morphologic information on cells at the fourth passage of culture. Panels (**A**–**C**): morphology, intracellular glycogen deposits, and organelles of URECs from a GSD1a patient; panels (**D**–**F**): morphology, organelles, and cilia of URECs from a control subject.

At the fourth passage, URECs from GSD1a subjects (Figure 1, Panels A–C) maintain their massive glycogen content, as detected by TEM (Figure 1, Panel A). No sign of cellular stress was seen compared to cells from healthy volunteers (Figure 1, Panels D–F), which were cultured in the same experimental conditions. At this passage, URECs presented the ciliary structures of the renal tubular epithelium (Figure 1, Panel D). After a first difference in the time needed to establish the initial URECs colonies, with less time observed in the GSD1a as compared to healthy control URECs (mean 12.6 vs. 17 days), at the following splitting passages, the number of days needed by the cells to become confluent and to be split into a 1:2 ratio was similar (mean: 5 days for both URECs groups).

**Figure 2:**
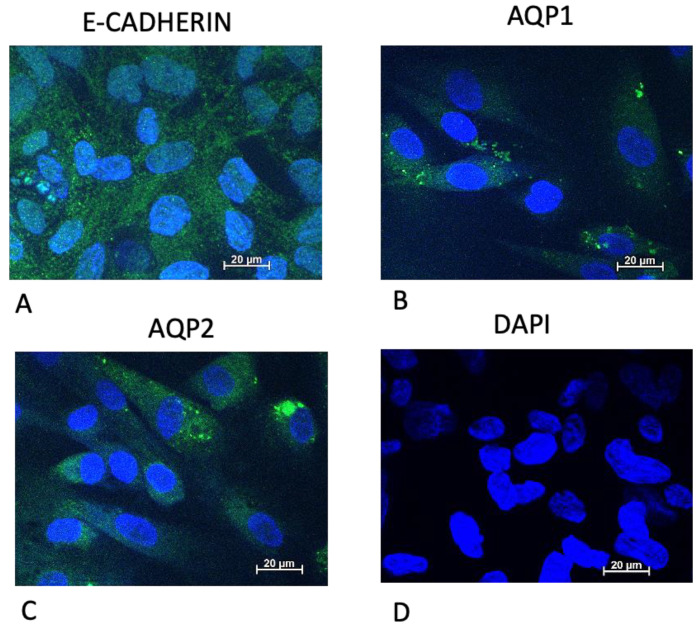
Immunofluorescence microscopy of GSD1a URECs stained with E-cadherin (green, (**A**) panel), an adherens epithelial junctions marker; with Aquaporin-1 (AQP1, green, (**B**) panel), a kidney proximal tubule marker; with Aquaporin-2 (AQP2, green, (**C**) panel), a collecting duct marker. Cell nuclei were counterstained with DAPI (blue, (**D**) panel). Scale bars, 20 µm.

To address the properties of epithelial monolayers, we tested the expression of adherens epithelial junctions (E-cadherin) and markers of tubular origin (aquaporin 1 and 2). The staining was positive for all the markers in both healthy control (not shown) and GSD1a (Figure 2, green signal) URECs; as the protocol was not selective for specific nephron segments, we obtained a mixture of kidney epithelial cells, as demonstrated by the co-existence of cells expressing aquaporin 1 (panel B) or 2 (panel C).

### 2.2. Analysis of ROS in GSD1a UREC

**Figure 3:**
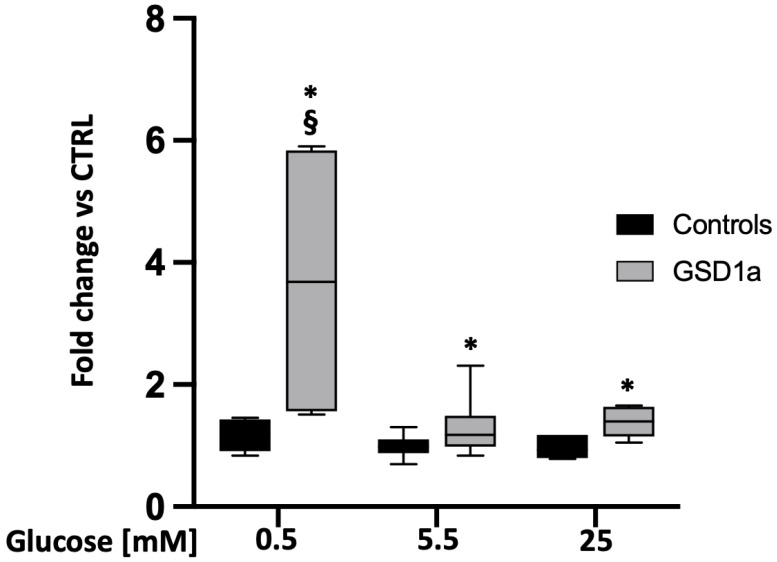
Quantification of reactive oxygen species (ROS) levels in URECs from GSD1a patients (*n* = 3) and control subjects (*n* = 3). ROSs were measured after 24 h of incubation of cells at different glucose conditions: low (0.5 mM), physiological (5.5 mM), and high (25 mM) glucose concentrations. The values obtained are reported as the mean ± SD of at least 3 technical replicates. Statistical significance was verified by unpaired *t*-test. * *p* < 0.05 GSD1a cells vs. control cells; § *p* < 0.001 GSD1a cells at low glucose (0.5 mM) vs. GSD1a cells at physiological (5.5 mM) and high (25 mM) glucose.

By using the fluoroprobe DCFDA to measure ROS levels, we found that GSD1a URECs (*n* = 3) had higher ROS levels (Figure 3): fold change vs. control: 3.7 ± 0.9, *p* = 0.03 at low glucose (0.5 mM); fold change vs. control: 1.3 ± 0.1, *p* = 0.007 at physiological (5.5 mM) glucose; and fold change vs. control: 1.4 ± 0.1, *p* = 0.01 at high glucose (25 mM). At low glucose, cells from GSD1a patients had higher levels of ROS (*p* < 0.001) compared to GSD1a cells cultured at physiological (5.5 mM) and high (25 mM) glucose (Figure 3).

## 3. Discussion

In this study, we showed, for the first time, that URECs from patients with GSD1a could be cultured until the fourth passage, maintaining their biological features. In fact, we found that they continue accumulating glycogen, present markers (e-cadherin, aquaporin 1 and 2), and structures (cilia) specific to renal epithelial cells.

We chose to set up this ex vivo human model to study GSD1a because URECs are easily collectible in a non-invasive way. Moreover, as kidney disease is a common complication observed in GSD1a, URECs represent a suitable source of cells for studying renal epithelial cell functions in this genetic condition. This cell model can be used in other rare metabolic diseases characterized by a specific and well-defined kidney phenotype for these advantages [14,15,16].

As the first proof-of-concept study, we tested the hypothesis that URECs from patients with GSD1a could be an experimental model to study the effects of pathological glycogen storage at the variation of a metabolic stimulus (glucose concentrations) on ROS levels in renal epithelial cells. We focused on ROS because an increase of kidney oxidative stress was reported in mice with renal-targeted deletion of G6Pase [6].

Functional characterization of these primary cells revealed distinct metabolic characteristics related to the genetics of GSD1a.

Even if we found that cells from both the GSD1a and control groups can respond to changes in glucose conditions by modulating ROS production, URECs from patients with GSD1a had higher levels of ROS in all glucose conditions as compared to control cells. Interestingly, the highest levels of ROS in GSD1a cells were detected at low glucose concentrations (0.5 mM), which is the condition that more resembles the metabolic derangement of patients with GSD1a, as these subjects are not able to physiologically restore euglycemia by metabolizing stored glycogen due to the inactivation of the G6Pase [1].

In conclusion, although the elucidation of the molecular mechanisms that cause the metabolic alterations of GSD1a in renal cells deserves further studies, the evidence of a disease-specific response of GSD1a URECs to a metabolic (low glucose) stimulus suggests that this cell model can be a promising tool for dissecting GSD1a nephropathy.

## 4. Materials and Methods

### 4.1. Preparation and Culture of UREC

Urine (100–200 mL) was collected, after informed consent from the subjects, in sterilized containers with 100 U/mL Penicillin, 100 µg/mL Streptomycin, and 2.5 µg/mL Amphotericin B, following the protocol described by Zhou et al. 2012 [17].

Due to the high risk of contamination, cells were isolated immediately after urine collection by centrifuging at 400× *g* for 10 min at room temperature. The supernatant was carefully aspirated, and 10 mL Phosphate Buffered Saline (PBS ECB400LX10 Euroclone) containing streptomycin/penicillin and amphotericin B was added to the samples, which were centrifuged at 400× *g* for 10 min. The supernatant was discarded, and 0.2 mL of the pellet was resuspended with 1 mL of primary medium (DMEM/Ham’s 12 Nutrient mix 1:1 (SH3027010 Euroclone, Pero-MI, Italy), supplemented with 10% Fetal Bovine Serum (FBS South America Origin EU ECS0180L Euroclone), 100 U/mL penicillin, 100 µg/mL Streptomycin (ECB3001D Euroclone), the REGM single Quot kit supplements (LOCC4127 Euroclone), and 2.5 µg/mL amphotericin B (ECM0009D Euroclone), and then transferred into a single well of 12 well plates coated with 0.1% of gelatin (wt/vol). Cells were incubated at 37 °C and 5% CO_2_ for 24 h. Every 24 h, 1 mL of primary medium was added to the cells. Three days after plating, the medium was aspirated, leaving approximately 1 mL, and then 1 mL of fresh medium was added and changed daily. When small colonies (four to five cells) appeared, the primary medium was changed with the proliferation medium every 2 days. Cells were expanded until 80–90% confluence and then split for further proliferation. Cells were resuspended in Cryostor (CS10 Voden, MI, Italy), then frozen at the fourth passage in liquid nitrogen for later use.

### 4.2. Morphological Analysis of Urine-Derived Renal Epithelial Cells

Transmission Electron Microscopy (TEM) was used to provide morphologic information on cells at the fourth passage of culture. Standard TEM protocols were used on a FEI Tecnai G2 microscope at the facility of the Department of Biology, University of Padova, Padova, Italy.

### 4.3. Immunofluorescence Protocol for URECs

We used a mouse anti-E-cadherin (610182 BD Biosciences, Milan, Italy), the polyclonal antibodies AQP1, Alexa Fluor®647 conjugated (BS-1506R-A647 CliniSciences, Nanterre France), and AQP2, Cy5 conjugated (BS-4611R-CY5 CliniSciences) to investigate cell origin.

URECs were seeded onto a round glass coverslip (Ø 13 mm) at a density of 1.0 × 10^4^ and incubated overnight with the proliferation medium. Different cells preparations were incubated with anti-E-cadherin or anti- AQP1 or anti-AQP2 at 4°C overnight. The primary antibody E-Cadherin was detected by incubation with anti-mouse IgG Alexa Fluor® 488 conjugated (Invitrogen, Carlsbad, CA, USA) for 1 h at room temperature.

Finally, cells were stained with 4,6-diamido-2-phenylindole (DAPI Sigma-Aldrich, St. Louis, MO, USA) for 15 min at 37°C for visualization of the nuclei. Images were acquired by using an Apotome.2 (Axiovert 200 M, Carl Zeiss MicroImaging GmbH, Göttingen, Germany) microscope.

### 4.4. Quantification of ROS

ROSs in URECs were measured by the Cellular ROS Assay kit (DCFDA/H2DCFDA-Cellular ROS Assay Kit AMab113851 Prodotti Gianni Srl Milan, Italy) according to the manufacturer’s instructions. Briefly, cells were seeded onto a dark, clear bottom 96 microplate with about 25,000 cells per well. Cells were incubated overnight at 37°C and 5% CO_2_. The next day, cells were cultivated for 24 h under normal (5.5 mM), high (25 mM), or low (0.5 mM) glucose conditions. Cells treated overnight with 100 µM H_2_O_2_ were used as a positive control. Then, cells were incubated with DCFDA for 45 min at 37°C and analyzed on a plate reader (Mithras LB 940 Berthold Italia Srl) at Ex/Em = 485/535 nm in endpoint mode. Each condition was performed at least in triplicate.

## Figures and Tables

**Table 1:** GSD1a patient characteristics. * Proteinuria was defined as excretion of protein/24 h > 0.15 g [13].

	Patient #1	Patient #2	Patient #3
Age (years)	40	44	29
Diagnosis	Enzimatic	Enzimatic	Biochemical
Gender	Male	Male	Male
G6Pase Mutations	p.R83C p.R170Q	p.R83C p.Q20R	NA
eGFR mL/min/1.73 m^2^	119	110	140
Proteinuria (g/24 h) *	No	No	Yes

## Data Availability

Not applicable.

## References

[1] Froissart R., Piraud M., Boudjemline A.M., Vianey-saban C., Petit F., Hubert-buron A., Eberschweiler P.T., Gajdos V., Labrune P. (2011). Glucose-6-Phosphatase Deficiency. Orphanet J. Rare Dis..

[2] Chou J.Y., Jun H.S., Mansfield B.C. (2010). Glycogen Storage Disease Type I and G6Pase-β Deficiency: Etiology and Therapy. Nat. Rev. Endocrinol..

[3] Yiu W.H., Pan C.J., Ruef R.A., Peng W.T., Starost M.F., Mansfield B.C., Chou J.Y. (2008). Angiotensin Mediates Renal Fibrosis in the Nephropathy of Glycogen Storage Disease Type Ia. Kidney Int..

[4] Visser G., Rake J., Labrune P., Leonard J., Moses S., Ullrich K., Wendel U., Smit P. (2002). Consensus Guidelines for Management of Glycogen Storage Disease Type 1b—European Study on Glycogen Storage Disease Type 1. Eur. J. Pediatr..

[5] Clar J., Gri B., Calderaro J., Birling M.C., Hérault Y., Smit G.P.A., Mithieux G., Rajas F. (2014). Targeted Deletion of Kidney Glucose-6 Phosphatase Leads to Nephropathy. Kidney Int..

[6] Yiu W.H., Mead P.A., Jun H.S., Mansfield B.C., Chou J.Y. (2010). Oxidative Stress Mediates Nephropathy in Type Ia Glycogen Storage Disease. Lab. Investig..

[7] Hijmans B.S., Boss A., van Dijk T.H., Soty M., Wolters H., Mutel E., Groen A.K., Derks T.G.J., Mithieux G., Heerschap A. (2017). Hepatocytes Contribute to Residual Glucose Production in a Mouse Model for Glycogen Storage Disease Type Ia. Hepatology.

[8] Lei K., Chen H., Pan C., Ward J.M., Mosinger B., Lee E.J., Westphal H., Mansfield B.C., Chou J.Y. (1996). Glucose-6-phosphatase dependent substrate transport in the glycogen storage disease type-1a mouse. Nat. Genet..

[9] Mutel E., Abdul-Wahed A., Ramamonjisoa N., Stefanutti A., Houberdon I., Cavassila S., Pilleul F., Beuf O., Gautier-Stein A., Penhoat A. (2011). Targeted Deletion of Liver Glucose-6 Phosphatase Mimics Glycogen Storage Disease Type 1a Including Development of Multiple Adenomas. J. Hepatol..

[10] Resaz R., Vanni C., Segalerba D., Sementa A.R., Mastracci L., Grillo F., Murgia D., Bosco M.C., Chou J.Y., Barbieri O. (2014). Development of Hepatocellular Adenomas and Carcinomas in Mice with Liver-Specific G6Pase-Aα Deficiency. DMM Dis. Model. Mech..

[11] Rutten M.G.S., Derks T.G.J., Huijkman N.C.A., Bos T., Kloosterhuis N.J., van de Kolk K.C.W.A., Wolters J.C., Koster M.H., Bongiovanni L., Thomas R.E. (2021). Modeling Phenotypic Heterogeneity of Glycogen Storage Disease Type 1a Liver Disease in Mice by Somatic CRISPR/CRISPR-Associated Protein 9-Mediated Gene Editing. Hepatology.

[12] Katagami Y., Kondo T., Suga M., Yada Y., Imamura K., Shibukawa R., Sagara Y., Okanishi Y., Tsukita K., Hirayama K. (2020). Generation of a Human Induced Pluripotent Stem Cell Line, BRCi009-A, Derived from a Patient with Glycogen Storage Disease Type 1a. Stem Cell Res..

[13] Kidney Disease: Improving Global Outcomes and CKD Work Group (2013). KDIGO 2012 Clinical Practice Guideline for the Evaluation and Management of Chronic Kidney Disease. Kidney Int..

[14] Ziegler W.H., Lüdiger S., Hassan F., Georgiadis M.E., Swolana K., Khera A., Mertens A., Franke D., Wohlgemuth K., Dahmer-Heath M. (2022). Primary URECs: A Source to Better Understand the Pathology of Renal Tubular Epithelia in Pediatric Hereditary Cystic Kidney Diseases. Orphanet J. Rare Dis..

[15] Schumann A., Belche V., Schaller K., Grünert S.C., Kaech A., Baumgartner M.R., Kölker S., Hannibal L., Spiekerkoetter U. (2021). Mitochondrial Damage in Renal Epithelial Cells Is Potentiated by Protein Exposure in Propionic Aciduria. J. Inherit. Metab. Dis..

[16] Luciani A., Schumann A., Berquez M., Chen Z., Nieri D., Failli M., Debaix H., Festa B.P., Tokonami N., Raimondi A. (2020). Impaired Mitophagy Links Mitochondrial Disease to Epithelial Stress in Methylmalonyl-CoA Mutase Deficiency. Nat. Commun..

[17] Zhou T., Benda C., Dunzinger S., Huang Y., Ho J.C., Yang J., Wang Y., Zhang Y., Zhuang Q., Li Y. (2012). Generation of Human Induced Pluripotent Stem Cells from Urine Samples. Nat. Protoc..

